# Medication Dispensing Patterns Among Individuals With Serious Mental Illness Using a Remote Medication Dispensing and Adherence Monitoring Platform: Cohort Study

**DOI:** 10.2196/79241

**Published:** 2026-09-02

**Authors:** George Unick, Nicole Mattocks, Cheuk Yui Yeung, Naomi Swenson, Karen Hopkins, Caitlin Manleigh

**Affiliations:** 1 School of Social Work University of Maryland, Baltimore Baltimore, MD United States; 2 Colorado Mesa University Grand Junction, CO United States; 3 Altruix, Inc Annapolis, MD United States

**Keywords:** medication adherence, telemedicine, community mental health services, telehealth, remote medication dispensing

## Abstract

**Background:**

Medication adherence is poor among individuals with serious mental illness (SMI). Few studies have demonstrated the effectiveness of remote medication dispensing and adherence monitoring interventions among individuals with SMI.

**Objective:**

This study aimed to understand medication dispensing rates for users of a remote medication dispensing and adherence monitoring device and to identify associated demographic and clinical characteristics.

**Methods:**

In this cohort study, individuals’ characteristics were measured at baseline, and dispensing records were followed from their enrollment and subsequent device installation as early as January 2019 until June 2023. Individuals were eligible to participate if they had an SMI diagnosis, were aged 18 to 64 years, were currently being prescribed psychiatric medications, and were receiving mental health services from a participating community mental health agency. Participants were recruited through a combination of self-selection and referrals from agency staff. Our intervention involved using a remote medication dispensing and adherence monitoring device to measure participants’ daily medication dispensing.

**Results:**

The final sample consisted of 99 participants. The mean age of the participants was 49 (SD 12.08) years; 64% (n=63) of the participants identified as men and 41% (n=41) as Black or African American. The overall dispensing rate was 92.9%, with 90 (91%) individuals having dispensing rates >80%. The results of the hierarchical Bayesian logistic regression model showed that participants adhered better to evening doses than morning doses (incidence rate ratio [IRR] 1.11, 95% credible interval [CrI] 1.06-1.16). Dispensing adherence was poorer on weekends than on weekdays (IRR 0.87, 95% CrI 0.83-0.91). For every additional year of using the device, the rate of adherence increased by 1% (IRR 1.01, 95% CrI 1.00-1.01). The rate of dispensing dropped by 22% after the onset of the COVID-19 pandemic (IRR 0.78, 95% CrI 0.71-0.86), and African American participants had a 29% lower rate of dispensing than White participants (IRR 0.71, 95% CrI 0.55-0.90). The rate of dispensing did not differ by age; sex; educational attainment; or the level of sadness, emotional and behavioral dyscontrol, cognitive function, or psychotic symptoms at baseline.

**Conclusions:**

The high adherence rate observed, regardless of baseline psychopathology levels, highlights the potential of remote medication dispensing and adherence monitoring devices to address adherence challenges in people with SMI. Observed variation in dispensing behavior by dose timing and contextual factors suggests opportunities for intervention, including aligning dosing schedules with patient routines, providing additional support during periods of disruption (eg, weekends or major life events), and tailoring strategies to address disparities across patient groups. These findings highlight the role of targeted, context-aware approaches to improve adherence in community-based SMI care. These findings support the integration of digital adherence monitoring within mental health services, especially in settings where traditional adherence support may be challenging.

**Trial Registration:**

ClinicalTrials.gov NCT03775044; https://clinicaltrials.gov/study/NCT03775044

## Introduction

More than 1 in 20 individuals in the United States are diagnosed with a serious mental illness (SMI), such as schizophrenia, bipolar disorder, or depressive disorders [1]. These individuals are at risk for serious adverse psychiatric, somatic health, and social outcomes [2]. Individuals diagnosed with SMI have higher rates of cancer, heart disease, high blood pressure, obesity, and diabetes, as well as worse outcomes compared with the general population [3-19]. This disparity in health outcomes is not a necessary consequence of mental illness but is the result of modifiable health factors that are poorly treated due to the interaction between health systems, poverty, health behaviors, and unmet health-related social needs [20,21].

Key to addressing the problem of poor health outcomes for individuals diagnosed with SMI is the integration of behavioral and somatic health care, particularly providing medication adherence support, which is essential to controlling the symptoms and causes of chronic psychiatric and somatic morbidity and early mortality. Medication adherence is particularly challenging for individuals diagnosed with SMI because of poor social support, psychotic and affective symptom severity, cognitive functioning, and demographic factors such as age and sex [22]. Experts endorse good adherence as 80% or more of medication taken as prescribed, whereas the average patient diagnosed with schizophrenia or bipolar disorder takes 50% to 70% of prescribed somatic and psychiatric medications [23-25].

Helping individuals maintain adherence to psychiatric and somatic medications while supporting recovery and delivering patient-centered care has been a challenge for public behavioral health systems [26]. For the highest-cost users of mental health systems, community-based psychiatric residential rehabilitation programs have been developed to provide varying levels of intensive medication management and recovery support in group home or congregate living settings and have been shown to improve adherence [27]. Typically, these programs have staff members directly observe the consumer taking medication twice a day or more. Weekly medication packing, per-shift documentation of controlled medications, and regular documentation of observed ingestion through manual recording in Medication Administration Records are required by regulations [28]. These systems are labor intensive, expensive, error prone, and intrusive. They also fail to effectively support individuals in developing medication self-management skills, which are essential for transitioning to a lower, more cost-effective level of care [29].

Electronic medication use data provide an opportunity to improve health and psychiatric outcomes by enabling care providers to monitor adherence more accurately, objectively, and in real time. Studies suggest that the use of telehealth can improve medication adherence among individuals with SMI by facilitating communication between consumers and prescribers and by developing routine-based systems that reach individuals at home (where medications are taken daily) rather than in a clinic [30-32]. This is also useful when geographic distance between prescribers and consumers delays interventions, reducing the effectiveness of coordinated care on health outcomes [33]. Electronic monitoring is an objective reference standard for adherence assessment methods, increasing the accuracy and timeliness of the data [34,35]. Monitoring and feedback have been shown to enhance communication and transparency, strengthen client engagement, and improve outcomes independent of the specific treatment approach [35]. This contrasts with clinician assessments of adherence, consumer self-report, pill counts, or medication prescription refill activity, which are inherently unreliable and do little to advance adherence [12,23-25,36-38]. By automating the data collection in real time, remote medication dispensing and adherence monitoring interventions allow the data to be used when needed and can alert prescribers that consumers need immediate follow-up.

The purposes of this study were to (1) understand medication dispensing rates associated with use of a remote adherence device and (2) identify demographic and clinical characteristics that are associated with different medication dispensing rates.

## Methods

### Remote Medication Dispensing Device and Adherence Monitoring Platform

Medherent is a remote medication dispensing and adherence monitoring intervention platform designed for individuals with SMI living in the community. It is currently being used to dispense medication and monitor consumer adherence. It is a fully supported digital hub with an always-on network connection and provides a cost-effective mechanism for increased care coordination through real-time dispensed data and missed-dose alerts. The Medherent device is a “smart” vending machine small enough to be mounted on a wall in consumers’ residences, developed and designed to specifically provide medication to individuals who have difficulty adhering to their complex regimens. Each device is used for only 1 consumer, allowing customization of dosing schedules.

**Figure 1:**
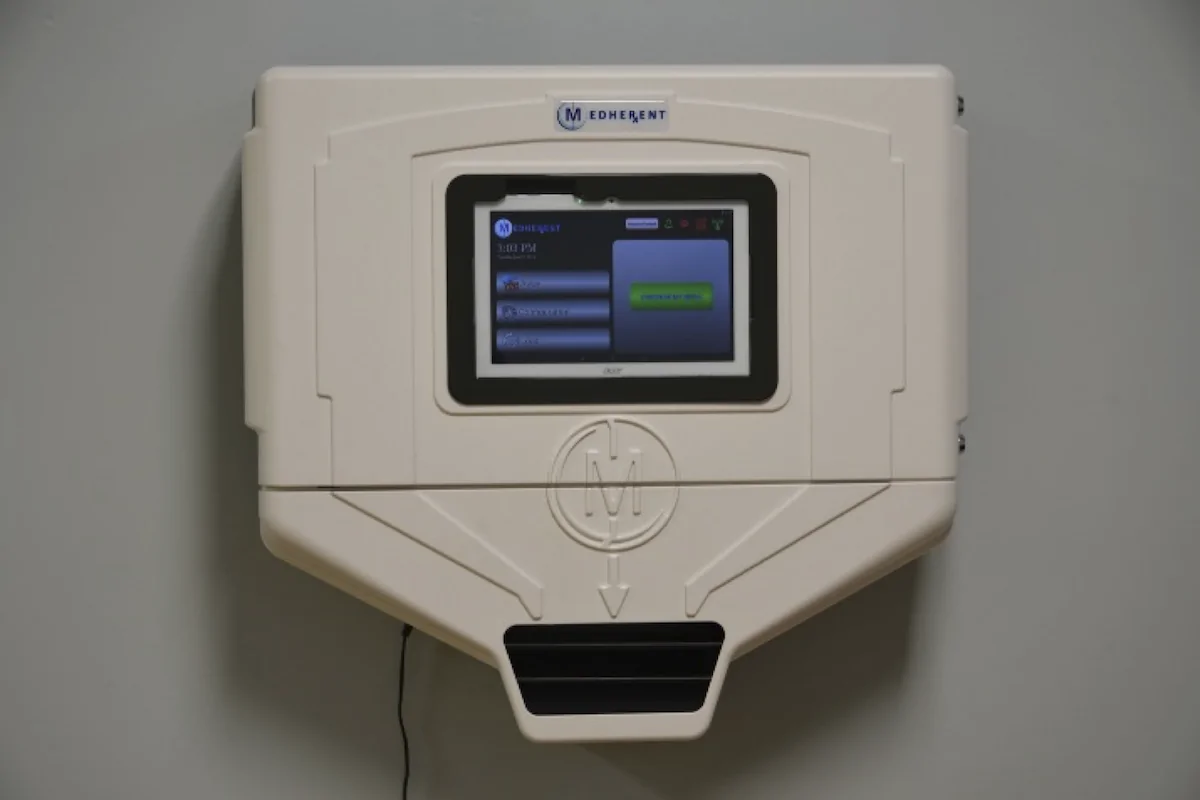
Medherent remote medication dispensing and adherence monitoring device.

Medherent currently automates the medication management process by providing a comprehensive, end-to-end adherence solution for consumers and care managers. Pharmacy staff load devices with a 14-day to 30-day- supply of the consumer’s oral medications prepared in multidose packets by the pharmacy’s robotic technology. The embedded Android tablet controls the device, is programmed with a unique user ID code, and receives downloads of the user’s current dosing schedule. Consumers receive audible and visual alerts when it is time to dispense their medication dose and can also receive SMS text messages. During the consumer’s scheduled 2-hour dosing window or until the unit is activated, a large green button reading “Dispense My Meds” remains on the Android screen (Figure 1). Users simply touch the button and enter a unique personal ID number to obtain the medications that correspond to that dosing time. Users are prompted by auditory and visual cues every 15 minutes until they activate the device. The tablet houses no protected health information and only incorporates the dosing schedule. The device’s activation populates an electronic medication record showing that the medication scheduled for that dosing time has been accessed by the assigned device user. Although Medherent does not confirm that the consumer has ingested the medication, evidence from other electronic monitoring devices, such as medication event monitoring system caps, provides evidence that dispensing is highly correlated with medication consumption [35,37,39].

The device is limited to oral medications that can be packaged into individualized, multidose packets. Medications prescribed in liquid, film, or injectable forms cannot be dispensed by the Medherent device. Additionally, individuals may access oral medications without the device recording dispensing. For example, the device can be opened by agency staff in the event of a malfunction. Individuals are not restricted from accessing medications outside of the device, depending on clinical and logistical circumstances. Consequently, device-recorded dispensing represents a lower bound of estimated adherence.

### Ethical Considerations

All procedures were approved by the University of Maryland, Baltimore (UMB) Institutional Review Board (HP-00083634), and participants were consented into the study following an assessment of their capacity to consent. All participants completed an institutional review board–approved informed consent process and a formal assessment of capacity to consent. This assessment required participants to accurately describe the study objectives and the specific details of their involvement to ensure full comprehension before enrollment. Informed consent and all subsequent participant interviews took place either at the agencies’ day programs or in participants’ private residences or group homes. The participants were informed that their participation in this study was entirely voluntary and would not affect the services they received from the agencies. All interviews were conducted in a private room in which only the client and interviewer were present, and US $20 incentives were provided for agreeing to participate in each interview.

### Participants and Procedures

Participants were recruited from 1 of 18 community mental health agencies (CMHAs) that agreed to work with the study team. The CMHAs were all publicly funded primarily through Medicaid and Medicare to provide mental health services, including residential rehabilitation, psychiatric rehabilitation, medication management, and other psychiatric rehabilitation services. Eligibility criteria for study participants included having a clinical diagnosis of serious and persistent mental illness (eg, charted diagnoses of schizophrenia, bipolar disorder, or major depressive disorder), currently being prescribed psychiatric medications, being aged between 18 and 64 years, and currently receiving mental health services from a CMHA. All participating CMHAs were using Altruix Pharmacy services for client medications at the time of the study. Participants were recruited through a combination of self-selection and referrals from agency staff. At each participating agency, pharmacy staff delivered presentations to CMHA staff and participants about the medication dispensing device.

In total, 161 participants receiving services from community mental health providers in Maryland consented to participate in this study. Participants were interviewed at the beginning of the study (baseline) to gather the following information related to demographics, medication adherence and health status. Instruments were selected based on the existing literature on clinical characteristics that affect adherence rates in individuals diagnosed with SMI [23].

### Demographics

Information regarding age, sex, race or ethnicity, and educational attainment was collected to characterize participants’ demographic characteristics.

### Medication Adherence

The Domains of Subjective Extent of Nonadherence is a brief, validated 3-item self-report measure of nonadherence [40]. Items assess missing or skipping medication doses. Consistent with recommendations, nonadherence is scored as the average of the 3 items.

### Health Status

The Prodromal Psychotic Symptom Questionnaire–Brief version was used to measure psychotic symptoms [41]. This questionnaire consists of 21 items. An 8-item measure of health-related quality of life for clinical research in neurology, Quality of Life in Neurological Disorders Item Bank 1.0–Short Form, was used to evaluate emotional and behavioral dyscontrol [42]. Quality of Life in Neurological Disorders Item Bank 2.0 Cognitive Function––Short Form, with 8 items, was used to assess perceived cognitive problems. The National Institutes of Health’s Patient-Reported Outcomes Measurement Information System–Sadness Short Form, with 8 items, was used to assess patient-reported sadness [43].

### Dispensing Data

**Figure 2:**
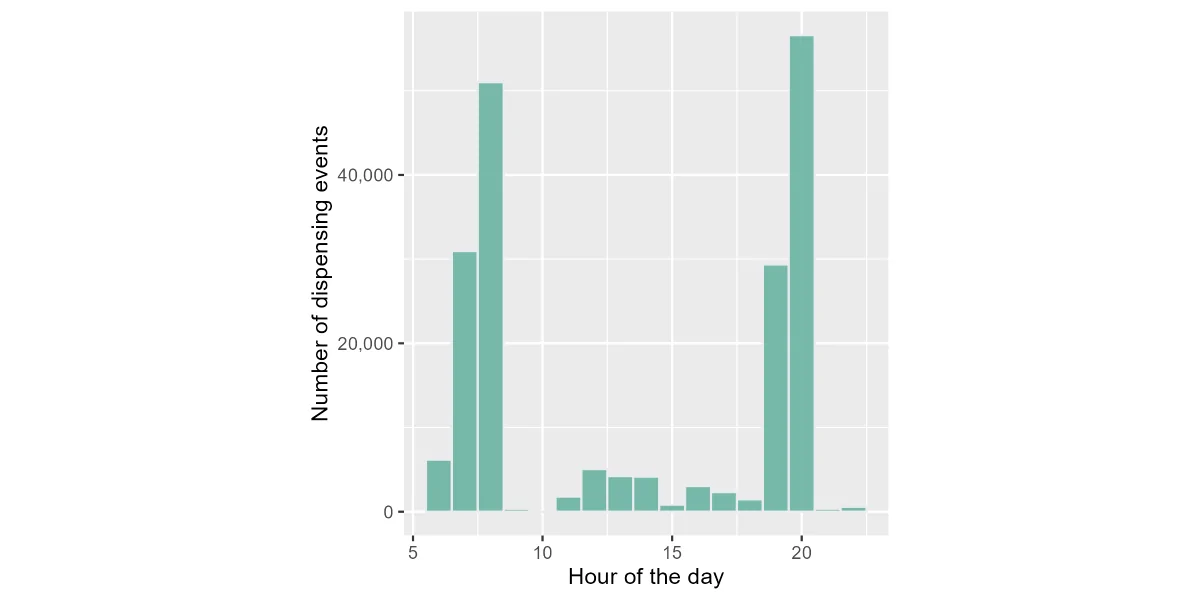
Counts by dispensing time.

The records of medication dispensing by the device for each individual were retrieved from their first use (as early as January 23, 2019) through June 23, 2023. Doses dispensed between 6 AM and 10 AM were considered as “morning doses,” and those dispensed between 6 PM and 10 PM were considered as “evening doses.” More than 80% of medications were dispensed in these periods (Figure 2). Afternoon doses were prescribed less frequently and often included medications that could be taken as needed (ie, pro re nata). Because these doses are often prescribed as needed, they do not reliably indicate dispensing behavior, and we excluded them from the analysis. Because individuals can skip doses dispensed by their medication dispensing device for reasons other than nondispensing, including hospitalization, vacation, or medication changes that require use of nonoral medications, we excluded missed doses for any consecutive nondispensing period of 14 doses or more (ie, 7 days). We assume that more than 1 week of consecutive missed doses in these supervised residential facilities is due to reasons other than nondispensing, and our discussions with agencies suggest that these assumptions are strongly conservative.

### Data Analysis

We estimated a hierarchical Bayesian logistic regression model using the Dynamite package (version 1.6.2), which leverages Stan for Bayesian inference [44]. The outcome variable was whether the medication was dispensed (1) or not dispensed (0) within a 2-hour window of the scheduled dose. Dispensing was modeled as a Bernoulli-distributed binary response, predicting the probability of success at each time point (eg, morning or evening). The model incorporated 2 autoregressive lags to account for temporal dependencies between subsequent doses (eg, a morning dose followed by the evening dose on the same day) and alternating doses (eg, subsequent morning doses).

Key individual-level predictors included measures of sadness, emotional and behavioral dyscontrol, cognitive function, psychotic symptoms, and self-reported medication adherence. Additionally, sociodemographic and contextual factors were included, such as African American race, less than high school education, age, female sex, weekend indicator, time using the medication dispensing device, afternoon dose, and the COVID-19 period, with the onset of the pandemic set as March 5, 2020.

To capture individual variation in dispensing trajectories, we incorporated random intercepts to account for within-individual correlations in repeated-measures data, as well as random slopes for time (half-day) to model changes over time. Additionally, natural splines (*df*=10) were used to flexibly capture nonlinear temporal trends. The model was estimated using 4 Markov Chain Monte Carlo (MCMC) chains, with parallel computation across 4 cores.

This Bayesian approach provides posterior distributions for all parameters, allowing robust estimation and uncertainty quantification while accounting for complex temporal and individual-level dependencies. The analysis was conducted in R software (version 4.3.2; R Foundation for Statistical Computing) with the package “dynamite” [45].

## Results

### Participant Characteristics

**Figure 3:**
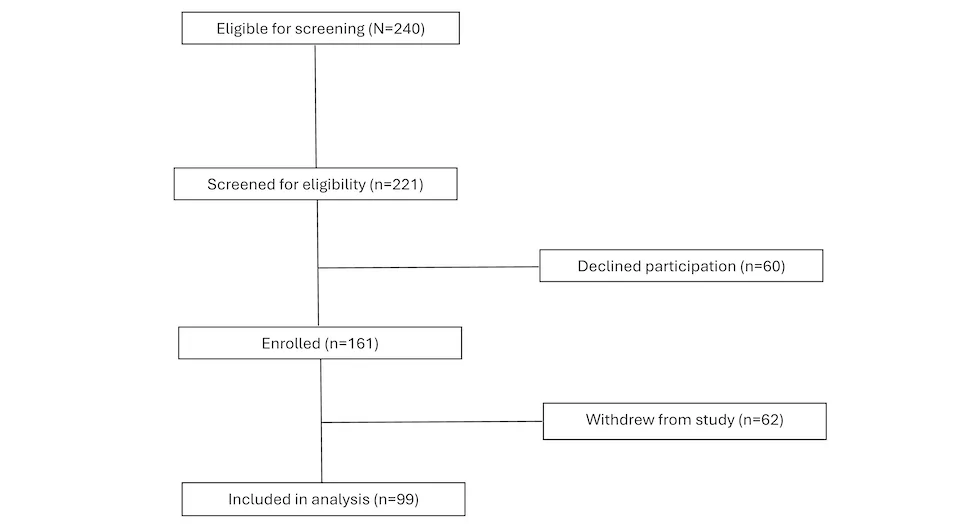
Flow diagram for study participants.

Figure 3 shows the recruitment process. On the basis of the eligibility criteria discussed previously, there were 221 individuals eligible to participate in the study, and 161 (73%) of them enrolled in the study. In total, 62 (39%) recruited participants were excluded from the analysis because their devices were not installed, resulting in no data collection. The reasons for noninstallation included agencies withdrawing due to COVID-19 precautions, participants leaving their residences or agency, and participant withdrawal from the study before devices were installed. The final sample consisted of 99 (61%) participants, with a total of 125,198 dispensing records. The median number of days using the medication dispensing device was 830 (IQR 266.5-1278.5) days. Participants included and excluded from the final sample did not show statistically significant differences in their baseline characteristics (Table 1).

**Table 1:** Characteristics of participants included in and excluded from the analysis of remote medication dispensing and adherence monitoring devices.

Characteristics		Participants included (n=99)	Participants excluded (n=62)	*P* value	
Age (years), mean (SD)		48.98 (12.08)	48.15 (12.49)	.67	
**Sex, n (%)**					.74
	Male	63 (63.64)	41 (66.13)		
	Female	36 (36.36)	21 (33.87)		
Black or African American, n (%)		41 (41.41)	35 (56.45)	.06	
Highest educational attainment: high school or below, n (%)		23 (23.23)	15 (24.19)	.89	
**Health status questionnaires, mean (SD)**					
	Sadness (Patient-Reported Outcomes Measurement Information System–Sadness short form)	17.24 (7.06)	16.60 (7.52)	.59	
	Emotional and behavioral dyscontrol (Neuro-QoL^a^)	16.34 (6.59)	15.84 (6.59)	.64	
	Cognitive function (Neuro-QoL version 2.0)	29.23 (5.72)	29.00 (6.49)	.81	
	Psychotic symptoms (Prodromal Questionnaire––Brief)	0.85 (1.85)	0.90 (1.68)	.85	
	Self-reported medication nonadherence (Voils Adherence Scale)	19.47 (9.05)	19.00 (8.30)	.74	

^a^Neuro-QoL: quality of life in neurological disorders.

The baseline characteristics of the participants are shown in Table 1. The mean age of the participants was approximately 49 (SD 12.08) years; 64% (n=63) of the participants identified as men and 41% (n=41) as Black or African American.

### Dispensing Rates

The overall rate of dispensing across all participants during the study period was 92.9%. The range of participant-level average dispensing rates was 44% to 100%, with 90 (91%) of the 99 individuals having dispensing rates greater than 80%. It is important to note that participants can take their medications outside of the medication dispensing device, so these estimates represent a lower bound of adherence.

### Predictors of Dispensing

Using the Dynamite R wrapper for Stan, we estimated a model predicting dispensing by demographic and clinical characteristics. After fitting the model, we examined MCMC convergence. The MCMC diagnostics indicate that the final model performed well overall, with no divergences, excessive tree depths, or low energy-Bayes fraction of missing information, suggesting stable and efficient sampling. Effective sample size (ESS) values were generally adequate, although the smallest bulk-ESS was 250 for 1 variance parameter, indicating some autocorrelation and slightly weaker mixing for that parameter. Most other bulk-ESS and tail-ESS values exceeded 750 and 1300, respectively, reflecting satisfactory sampling efficiency overall. The R̂ convergence diagnostic values were close to 1.00, with the highest at 1.02, suggesting near convergence with minor residual variability. Overall, the model demonstrates acceptable convergence diagnostics, and the model appears to be performing reliably.

Model results (Table 2) indicate that dispensing was poorer on weekends than on weekdays (incidence rate ratio [IRR] 0.87, 95% credible intervals [CrIs] 0.83-0.91). Compared to morning doses, participants adhered better to evening doses (IRR 1.11, 95% CrI 1.06-1.16). For every additional year on the device, the rate of dispensing increased by 1% (IRR 1.01, 95% CrI 1.00-1.01). The rate of dispensing dropped by 22% (IRR 0.78, 95% CrI 0.71-0.86) after the onset of the COVID-19 pandemic. African Americans participants had a 29% lower rate of dispensing compared to White participants (IRR 0.71, 95% CrI 0.55-0.90). The rate of dispensing did not differ by age, sex, or educational attainment. There was no evidence that dispensing was related to the level of sadness, emotional and behavioral dyscontrol, cognitive function, or psychotic symptoms at baseline.

**Table 2:** Regression incidence rate ratios (IRRs) with 95% credible intervals (CrIs) for medication dispensing adherence.

Variable	IRR	95% CrI
Age (years)	1.00	0.99-1.01
Sex: female	1.11	0.86-1.42
Race: African American	*0.71* ^a^	*0.55-0.90*
Education: high school or below	0.93	0.68-1.23
Sadness	0.93	0.79-1.10
Emotional and behavioral dyscontrol	1.09	0.92-1.31
Cognitive function	0.94	0.79-1.23
Psychotic symptoms	0.85	0.78-1.14
Self-reported medication nonadherence	0.86	0.73-1.03
Day of the week: weekend	*0.87*	*0.83-0.91*
Evening dose	*1.11*	*1.06-1.16*
Year on adherence device	*1.01*	*1.00-1.01*
COVID-19 pandemic	*0.78*	*0.71-0.86*

^a^Italicization represents IRR values that have 95% CrIs that do not cross 1, consistent with *P*<.05.

**Figure 4:**
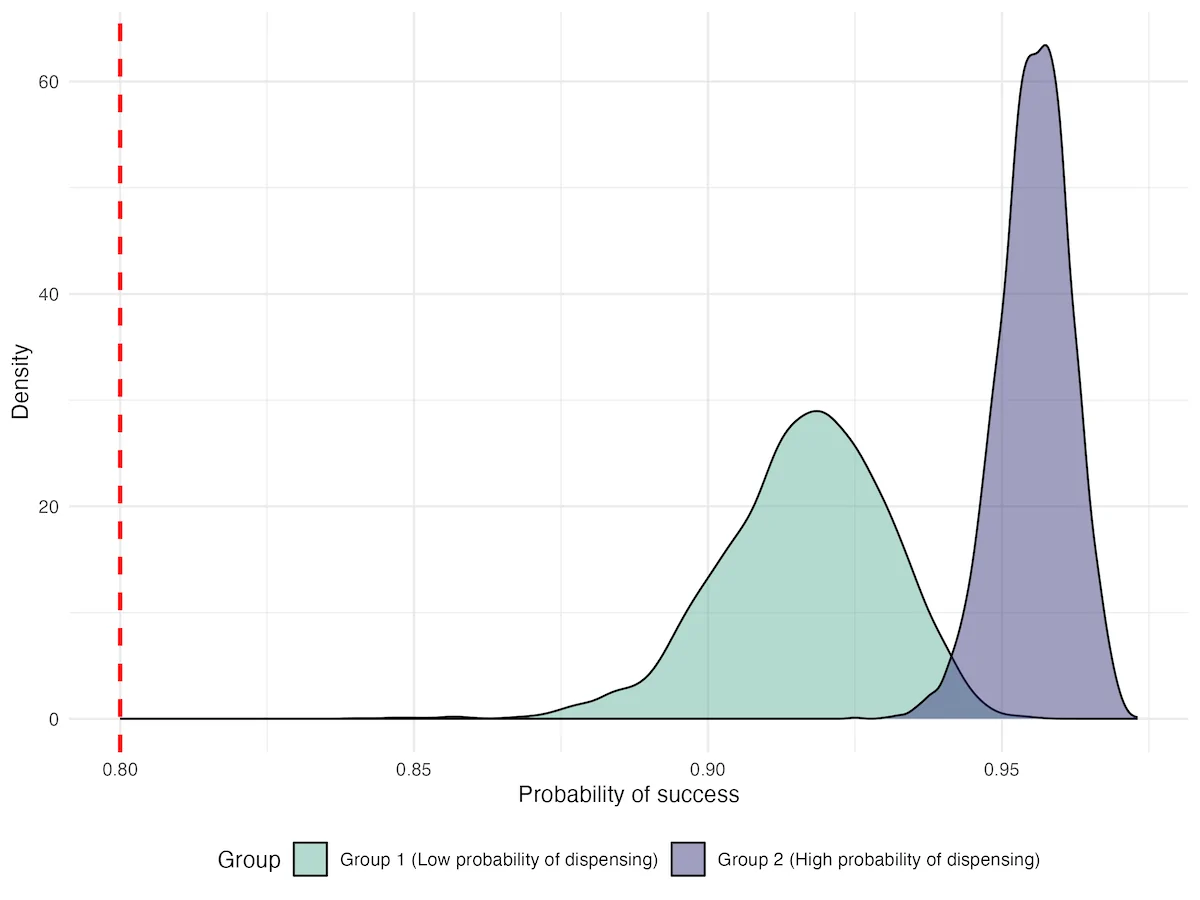
Comparison of predicted probability of medication dispensing.

To demonstrate the model-implied predicted probability of dispensing, we used the posterior distribution to estimate the probability of dispensing for 2 groups: a low-probability dispensing group and a high-probability dispensing group (Figure 4). The low-probability group was defined as a male African American with average values for clinical variables, such as depression and psychotic symptoms, who dispensed their morning dose of medication on the weekend after the start of the COVID-19 pandemic. The high-probability group was defined as a White female participant with average values for clinical variables, such as depression and psychotic symptoms, who dispensed her evening dose of medication on a weekday before the start of the COVID-19 pandemic. As shown in Figure 2, both groups had a greater than 90% probability of dispensing their medication, well above the 80% adherence cutoff.

## Discussion

### Principal Results

This study aimed to evaluate medication dispensing in individuals with SMI using the remote medication dispensing and adherence monitoring intervention, examining adherence rates and factors influencing dispensing patterns. Overall, the dispensing rates observed were high across all groups, with an average dispensing rate of 90%, underscoring the effectiveness of the system in promoting consistent access to medication.

The clinical utility of these findings includes the specific contextual factors associated with increased medication dispensing, such as evening dosing and the duration of device use. Clinicians working with people with SMI may benefit from these results by prioritizing the scheduling of critical medications for evening hours, during which our data showed significantly higher dispensing rates. Furthermore, the finding that adherence likelihood increased with years on the medication dispensing system, although modestly, suggests a longitudinal “habituation effect,” where the device becomes an integrated part of the user’s daily ecology over time.

Importantly, our finding that medication dispensing with the device was not associated with baseline clinical symptoms, including cognitive impairment, sadness, or psychotic symptoms, suggests that the device can be an effective tool for helping individuals diagnosed with SMI. Consistent with other research on adherence support systems, this finding suggests that the dispensing device’s automated, structured nature may effectively offload the cognitive burden of medication management [46]. This is particularly evident in our profile analysis; even individuals categorized in the “highest-risk” clinical profile achieved dispensing rates consistent with medical guidelines (typically 80%).

### Limitations

This study has several limitations. First, these analyses cannot confirm whether individuals actually took their medications; they only indicate that the medications were dispensed. Although future analyses will explore this question, our current findings are limited to describing dispensing patterns rather than actual adherence. Fortunately, evidence from the medication event monitoring system cap literature suggests a very strong correlation between dispensing and adherence [35,37,39]. Second, the current analysis did not include a control group for comparison between individuals who used the device and those who did not. Additionally, due to COVID-19 pandemic–related disruptions and attrition, some individuals recruited into the study never received a medication dispensing device. Although there were no significant differences in demographics or baseline characteristics between those who received the device and those who did not, there remains a potential for attrition-related bias. Furthermore, these findings are limited to the scheduled morning and evening doses and do not reflect the use of pro re nata medications or medications that are scheduled outside of the morning and evening dosing windows and may not fully represent adherence to clinical intent or medication regimens. As noted, individuals may be prescribed liquid, film, or injectable medications that are not dispensed by the device, and they may also access oral medications outside of the device. Consequently, dispensing data do not capture the full range of prescribed medications and should be interpreted as an incomplete measure of adherence.

An important limitation is the representativeness of our sample; several methodological factors may have excluded the most marginalized segments of the population with SMI. The requirement for a stable home environment with a power source and cellular or Wi-Fi connectivity inherently filtered out individuals experiencing transient housing or extreme environmental instability. Future iterations of this research must focus on expanding the infrastructure to include those in supportive housing or transitional settings to ensure that the benefits of digital adherence tools reach the full spectrum of the SMI community. A related limitation is that all individuals in this sample received clinical support through residential rehabilitation housing or assertive community treatment teams that provided direct care. Future research should examine how the medication dispensing device interacts with these clinical and social support systems and how this interaction influences medication dispensing and adherence. Finally, this research was supported by the National Institutes of Health through the Small Business Innovation Research grant mechanism (award R44 MH116765-01) awarded to Altruix Pharmacy, the developer of the Medherent device. In accordance with UMB conflict of interest policies, a formal management plan was implemented to preserve the objectivity of the research. Although all data analysis and editorial decisions were conducted by UMB researchers and in full compliance with institutional oversight, we recognize that industry-sponsored research may carry an inherent risk of perceived bias. Consequently, independent verification through future replication studies by external research groups remains essential to confirm these findings and establish the generalizability of the Medherent system across diverse clinical settings.

### Comparison With Prior Work

These findings align with previous research suggesting that remote medication dispensing and adherence monitoring interventions can improve adherence by enhancing routine and offering real-time reminders, which are particularly valuable for individuals with complex medication regimens, psychiatric symptoms, and cognitive impairments [47-50].

### Conclusions

Interestingly, there were a few significant differences in dispensing based on participants’ levels of psychopathology. Dispensing behavior was statistically similar across baseline levels of sadness, emotional and behavioral dyscontrol, cognitive function, and psychotic symptoms. This lack of differentiation implies that the medication dispensing system may effectively mitigate barriers to adherence commonly associated with these factors, providing a stable support system irrespective of baseline psychological status. This suggests that even individuals with higher levels of psychological distress can maintain adherence when provided with structured, automated adherence tools.

Some dispensing variation did emerge based on contextual factors. Dispensing was generally lower on weekends and for morning doses. Several factors may contribute to this pattern. The reduction in dispensing following the COVID-19 pandemic suggests that external factors can still impact dispensing, although the medication dispensing device’s support may have helped mitigate some pandemic-related disruptions.

The high dispensing rate observed among participants, regardless of psychopathology levels, highlights the potential of remote medication dispensing and adherence monitoring tools to address adherence challenges in individuals with SMI. These findings support the continued integration of digital adherence monitoring within mental health services, especially in community-based settings where traditional adherence support may be challenging to implement consistently. Future research should further explore the factors contributing to racial and contextual disparities in adherence to optimize intervention design and improve equity in treatment outcomes.

## Abbreviations

CMHA: community mental health agency
CrI: credible interval
ESS: effective sample size
IRR: incidence rate ratio
MCMC: Markov Chain Monte Carlo
SMI: serious mental illness
UMB: University of Maryland, Baltimore
